# Genitourinary defects, anxiety and aggressive-like behavior and glucose metabolism disorders in *Zmym2* mutant mice with inserted piggyBac transposon

**DOI:** 10.3389/fcell.2025.1523266

**Published:** 2025-04-17

**Authors:** Rufeng Dai, Ye Yin, Minghui Yu, Yumeng Zhang, Jingjia Zhang, Tianyi Liu, Xiaoyan Fang, Xiaohui Wu, Qian Shen, Hong Xu

**Affiliations:** ^1^ Department of Nephrology, Children’s Hospital of Fudan University, Shanghai Kidney Development and Pediatric Kidney Disease Research Center, Shanghai, China; ^2^ State Key Laboratory of Genetic Engineering and National Center for International Research of Development and Disease, Institute of Developmental Biology and Molecular Medicine, Fudan University, Shanghai, China; ^3^ National Key Laboratory of Kidney Diseases, People's Liberation Army General Hospital, Beijing, China

**Keywords:** ZMYM2, piggyBac mice, genitourinary defects, anxiety and aggressive-like behavior, glucose metabolism disorders, Tbx18

## Abstract

Mutations in *ZMYM2* lead to syndromic congenital anomalies of the kidney and urinary tract (CAKUT) in humans. Tbx18 is co-expressed with Zmym2 in mesenchymal compartment of developing mouse ureter, indicating a potential *in vivo* relevance of the TBX18–ZMYM2 protein interaction in ureter development. The presence of multiple phenotypes beyond the urinary system in CAKUT patients carrying *ZMYM2* mutations suggests that ZMYM2 has extensive roles in various developmental processes. This study aims to comprehensively examine the multi-phenotypic consequence of *ZMYM2* mutations, with a particular focus on the roles of ZMYM2 in embryonic development, late metanephros formation, and the reproductive, nervous and endocrine systems, in addition to its role in urinary system. Using a new *Zmym2* mutant mouse model with an inserted piggyBac transposon (PB), we found that homozygous *Zmym2* mutations resulted in severe growth retardation of embryos by embryonic day 9.5 (E9.5D) and lethality from E10.5D. Heterozygous mutations caused morphogenetic issues in the genitourinary system, including duplex kidneys, vesicoureteral reflux (VUR), and cryptorchidism. And these heterozygous mutants exhibited anxiety and aggressive-like behaviors, and glucose metabolism disorders. Additionally, *Zmym2* mutations induced duplicated ureteric bud (UB) eruption and abnormal nephrogenic zone extension, contributing to duplex kidney formation. Reduced apoptosis in the nephric duct might have contributed to abnormal ureter-bladder connections, which could explain the observed cases of VUR. Notably, Tbx18 is co-expressed with Zmym2 in mouse kidney, reduced Tbx18 expression in *Zmym2* mutants further supports the hypothesis that *Zmym2* interacts with Tbx18 during kidney development. *Zmym2* PB mouse is the first model to demonstrate roles of *Zmym2* in neuroethology and endocrinology, extending its significant beyond genitourinary defects and embryonic development. Further investigation of these phenotypes in CAKUT patients carrying ZMYM2 mutations will enhance our understanding of their phenotypes and improve strategies for early diagnosis, monitoring, and treatment.

## 1 Introduction

Congenital anomalies of the kidney and urinary tract (CAKUT) are the most frequent birth defect (Loane et al., 2011; van der Ven et al., 2018a). Nearly 50% of chronic kidney disease (CKD) cases in children are caused by CAKUT, often progressing to end-stage kidney disease (ESKD) (Cirillo et al., 2023). Accurate diagnostic evaluation is pivotal to minimize kidney damage and prevent CKD. CAKUT encompasses a broad spectrum of phenotypes arising from defects in the development of urinary system. In murine models, the location and number of ureteric bud (UB) outgrowths and subsequent branching abnormalities lead to various CAKUT phenotypes, ranging from complete renal agenesis (RA) to more subtle defects, such as duplex or bifurcated ureters, vesicoureteral reflux (VUR), or other CAKUT subtypes (Blake and Rosenblum, 2014; Short and Smyth, 2020). Gene mutations or exposure to environmental risk factors disrupt the normal development processes, final, ultimately leading to CAKUT (Kolvenbach et al., 2023).

A substantial proportion of CAKUT cases have genetic basis. To date, approximately 54 monogenic genes have been identified as causative for CAKUT in humans, 135 genes are linked to monogenic multiorgan syndromes with facultative CAKUT (van der Ven et al., 2018b; Seltzsam et al., 2022). *ZMYM2* (MIM: 602221) encodes a nuclear zinc finger protein that localizes to the nucleus (Kunapuli et al., 2006), and is part of a transcriptional complex functioning as a corepressor by interacting with various nuclear receptors (Gocke and Yu, 2008). Our previous studies reported that the heterozygous truncating mutations in *ZMYM2* cause neurodevelopmental-craniofacial syndrome with variable renal and cardiac abnormalities (Connaughton et al., 2020). Multisystem phenotype caused by *ZMYM2* heterozygosity in humans suggests extensive further roles in development. The phenotypes of CAKUT patients carrying *ZMYM2* mutation may have been omitted due to insufficient assessment or a follow-up period that did not reach the age of onset of certain phenotypes.

The piggyBac (PB) transposon is a DNA transposon, that often disrupts gene expression upon insertion. The PB transposon mouse model serves as a valuable tool for studying CAKUT facilitating the investigation of pathogenic mechanisms, as well as the prevention, diagnosis and treatment of CAKUT (Ding et al., 2005; Wang et al., 2020). Using a newly generated *Zmym2* mutant mouse model with an inserted PB transposon, this study aims to comprehensively examine the multi-phenotypic consequences of Zmym2 mutations, with a particular focus on the roles of *Zmym2* in embryonic development, late metanephric formation, and the reproductive, nervous, endocrine systems, and urinary systems.

## 2 Materials and methods

### 2.1 Mice

**FIGURE 1:**
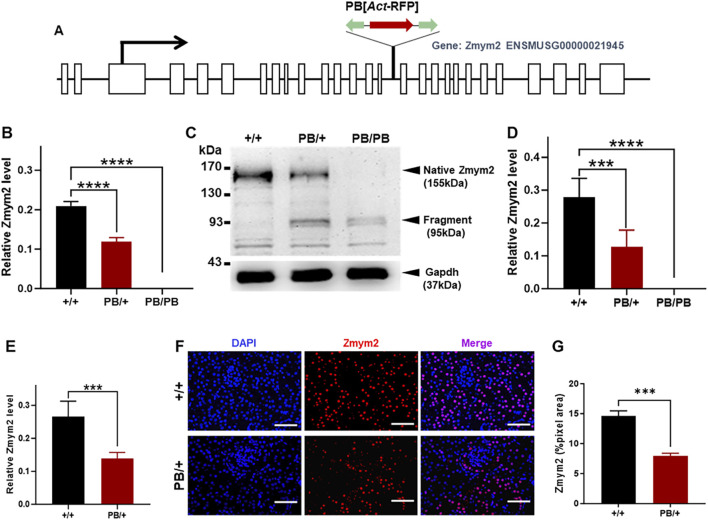
Characterization of the PB insertion in *Zmym2* mutant mice. **(A)** Genomic structure of the *Zmym2* PB allele. A PB [Act-RFP] transposon was inserted in the15th intron of Zmym2 on mouse chromosome 14, nucleotide 57,557,590. **(B)** After PB insertion, *Zmym2* mRNA level detected by Realtime-PCR in E9.5D embryos was decreased about 50% in *Zmym2* PB/+ group than that in *Zmym2* +/+ group, and there was no *Zmym2* mRNA expression in *Zmym2* PB/PB group. **(C, D)** Western blots showed that PB transposon insertion resulted in a significant truncated mutation in *Zmym2*. The protein expression level of Zmym2 in E9.5D embryo was decreased or there was no Zmym2 expression after PB insertion. **(E)** Zmym2 mRNA expression was approximately 50% reduced in *Zmym2* PB/+ kidney than that in *Zmym2* ^+/+^ kidney. **(F, G)** Zmym2 was mainly expressed in the nuclear of tubular cells, and weakly expressed in the cells of glomeruli, Scale bars = 50 μm. The quantification results of fluorescence average optical density also confirmed the expression of Zmym2 in *Zmym2* PB/+ mice is weaker than in *Zmym2* +/+ mice. ***, P < 0.001; ****, P < 0.0001.

All animal experiments were performed in accordance with protocols approved by the Animal Care and Use Committee of Children’s Hospital of Fudan University (NO. 2021-191). The *Zmym2* mutant strain (080214049-HLA) was established on the FVB/N background, and maintained on 12/12-h light/dark cycles. In the *Zmym2* PB allele, the PB insertion was mapped in the 15th intron of *Zmym2* (Chr:14.57557590, Ensembl ID: ENSMUSG00000021945) (Figure 1). FVB/N strain, Hoxb7-mVnus fluorescent mice were used. Hoxb7-EGFP plasmid was a generous gift from Professor Frank Constatini (Columbia University, New York City, NY) (Wang et al., 2018). When the mice were 6–8 weeks old, they were housed in the same cage with a female to male ratio of 3:1. The female mice were observed for pregnancy at 8:00 a.m. The earliest time when a vaginal plug was observed was counted as E0.5D. After the vaginal plug was observed, the female mice were randomly divided into two groups.

### 2.2 Genotyping polymerase chain reaction

The toes of neonatal mice at postnatal 7 days (P7D) or the yolk sac of E8.5D to E13.5D embryos were dissected from intercrossed *Zmym2* PB/+ female mice and used to extract genomic DNA, followed by genotyping polymerase chain reaction (PCR). Offspring with the transposon inserted into the *Zmym2* gene were genotyped using the primers P1 (5′-CTGAGATGTCCTAAATGCACAGCG-3′), P2 (5′-TCCAAAAGAGCTGGCATACTAAAGG-3′), P3 (5′-AGCACATTGTACTGTGTTCAGAGAG-3′). *Zmym2* PB/PB and *Zmym2* PB/+ were amplified by P1 and P3, which produced a 600 bp fragment. Wild type and *Zmym2* PB/+ were amplified by P2 and P3, which yielded an 864 bp fragment (Supplementary Figure S1). PCR conditions were as follows: initial denaturation at 93°C for 90 s; 40 cycles of 93°C for 30 s, 57°C for 30 s, and 65°C for 2 min; and a final extension at 65°C for 10 min. Genomic DNA extracted from mouse toes was used as template.

### 2.3 Gross phenotypic analysis

Phenotypes of *Zmym2* PB/+ and *Zmym2* +/+ mice were assessed. The animals were anesthetized with CO_2_.The location, morphology, and number of kidneys, ureters, and bladders of mice in each group were observed under a fluorescence stereoscope. The pregnant mice in difficult groups from E8.5D to E13.5D were anesthetized by CO_2_, the dissection was performed layer by layer, and the embryos were removed and observed. The kidney primordia were separated under a fluorescence stereomicroscope to observe the number and location of ND and UB. The images were recorded under a microscope (Leica, Germany).

### 2.4 RNA-sequencing

The RNeasy Mini kit (QIAGEN, Germany) was used to extract the total RNA of E9.5D embryos from the *Zmym2* PB/PB and *Zmym2* +/+ mice. The sequencing data was filtered with SOAP nuke (v1.5.2), afterward clean reads were obtained and stored in FASTQ format. The clean reads were mapped to the reference genome using HISAT2 (v2.0.4), Bowtie2 (v2.2.5) was applied to align the clean reads to the reference coding gene set, then expression level of gene was calculated by RSEM (v1.2.12). Using Fragments Per Kilobase of exon model per Million mapped fragments (FPKM) algorithm for the expression of standardized, which counts by total exon fragments/[mapped reads (millions) × exon length (kb)], reflects the gene expression level. Essentially, differentially expressed genes (DEGs) analysis was performed using the DESeq2 (v1.4.5) (Abdi, 2007) with fold change ≥2 and false discovery rate (FDR) ≤ 0.001. To take insight to the change of phenotype, GO1 enrichment analysis of annotated different expressed gene was performed by Phyper2 based on Hypergeometric test. The significant levels of terms and pathways were corrected by Q-value (=FDR) with a rigorous threshold (Q-value ≤0.05) by Bonferroni (Tan et al., 2021).

### 2.5 Immunofluorescence

The E12.5D kidney primordia were isolated and fixed in formaldehyde overnight. After washing with 0.3% Triton X-100/PBS, tissues were blocked by 5% donkey serum (Jackson) and placed on a shaker at 4°C overnight. The tissues were incubated with anti-Caspase 3 antibody (CST, #9662, 1:400), then placed in a shaker at 4°C overnight. The tissues were washed with PBS and incubated with Cy5-donkey anti-rabbit antibody (Jackson, 1:1,000). DAPI (1:1,000) was used to stain the nuclei. Six kidney primordia from each group were used, and the samples were mounted with 50% glycerol/PBS. Immunofluorescence done in the sections of kidney tissues was as previously described in the kidney primordia. Anti-Zmym2 antibody (NOVUS, #NB100-56490, 1:400) and Anti-Tbx18 antibody (4A Biotech, # ABIN522536, 1:400) were used as primary antibodies. Images were taken under a confocal microscope (Leica) and analyze date using ImageJ software.

### 2.6 Behavioral analyses

To evaluate anxiety, an open field test was conducted. Mice, approximately 8 weeks old, were acclimatized in the experimental room for over 1 h before the test began. The open field consisted of a 40 cm × 40 cm area enclosed by 40 cm high wooden walls painted white. Testing occurred between 13:00 and 17:00, with the arena cleaned between each mouse’s trial. Each mouse was placed in the center of the square, and its behavior was recorded on videotape for 15 min. The duration spent in both the peripheral zone and the center, as well as the distance traveled and instances of rearing in either area, were analyzed using a computerized technique with Etho Vision 3.0 (Noldus, Wageningen, Netherlands) (Walsh et al., 2017).

For the aggressive behavior test, the resident–intruder paradigm Koolhaas et al. (2013) was employed. Each male mouse was isolated for 1 h prior to testing and was evaluated in his home cage (acting as the resident) against a group-housed male intruder (four mice per cage). The behavior was recorded on video for up to 15 min. Key metrics included the latency to the first biting attack and the number of biting attacks during the subsequent 5 min following the first attack. If no biting behavior was observed for 10 min, the test was terminated, and the latency was recorded as 10 min. Testing was conducted between 12:00 and 16:00 and repeated three times for each male at 3-day intervals, ensuring that each male encountered a novel intruder during each trial. To assess locomotor activity, the duration of walking and the number of rearings were recorded for the 5 min following the first attack in the initial trial. If no biting behavior occurred, the latter 5 min of the 10-min period recorded was used to evaluate locomotor activity.

### 2.7 Glucose homeostasis

Blood glucose concentrations of 0-, 7-, 14-, 21-, 30-, 60-, 90- and180-day-old mice were measured using a glucometer, typically taken in either a random or fasted state. Oral glucose tolerance tests (OGTT) were performed in 90-day-old *Zmym2* PB/+ mutant mice and *Zmym2* +/+ littermate control mice as previously described (Kennard et al., 2021). Serum insulin concentrations in a random or fasted state and 30, 60, and 120 min after oral glucose application were quantified by ELISA (Abcam, #ab285341).

### 2.8 Statistical analysis

Numerical data are presented as the mean ± standard deviation. Statistical analysis was performed using GraphPad Prism version 10.0 and SPSS 20.0 statistical software. Two-sided unpaired t tests were used for between-group data, and χ2 tests were used for assessing count data. The significance level was set at p < 0.05, p > 0.05 representing no statistically significant distinction, all n = 6 at least in each group. In all figures, a single asterisk indicates p < 0.05, while double, triple, and quadruple asterisks indicate p < 0.005, p < 0.0005, and p < 0.0001, respectively.

## 3 Results

### 3.1 Genetic characterization of *Zmym2* PB mutant mice

The PB transposon was inserted into the 15th intron of *Zmym2* (Figure 1A). In E9.5D embryos, quantitative PCR analysis showed that *Zmym2* mRNA levels were reduced by nearly 100% in homozygous mutants (*Zmym2* PB/PB) compared to wild-type littermates (*Zmym2* +/+) and by approximately 50% in heterozygous mutants (*Zmym2* PB/+) (Figure 1B). Western blot analysis demonstrated that PB transposon insertion resulted in significant truncation of the Zmym2 protein. In the whole embryos tissues at E9.5D, a truncated protein band (95 kDa) was detected in both *Zmym2* PB/+ and *Zmym2* PB/PB lanes. Native Zmym2 protein (155 kDa) expressed in *Zmym2* PB/+ and *Zmym2* +/+ groups, and the protein level of native Zmym2 in *Zmym2* PB/+ group was reduced to approximately 50% of that in the *Zmym2* +/+ group. Notably, the native Zmym2 band is absent in the *Zmym2* PB/PB group (Figures 1C, D).

Furthermore, quantitative PCR analysis and immunofluorescence were performed to assess Zmym2 expression levels and distribution in the kidneys of mutant mice. *Zmym2* mRNA expression in *Zmym2* PB/+ kidneys was approximately 50% of that in *Zmym2* +/+ kidneys (Figure 1E). In kidney tissue sections, Zmym2 was predominantly localized in the nuclei of tubular cells, with weak expression detected in glomerular cells. Fluorescence quantification results were consistent with the quantitative PCR results, confirming that Zmym2 expression was lower in *Zmym2* PB/+ kidneys than that in *Zmym2* +/+ kidneys (Figures 1F, G).

### 3.2 *Zmym2* PB/PB mouse embryos show developmental abnormality and early lethality

**FIGURE 2:**
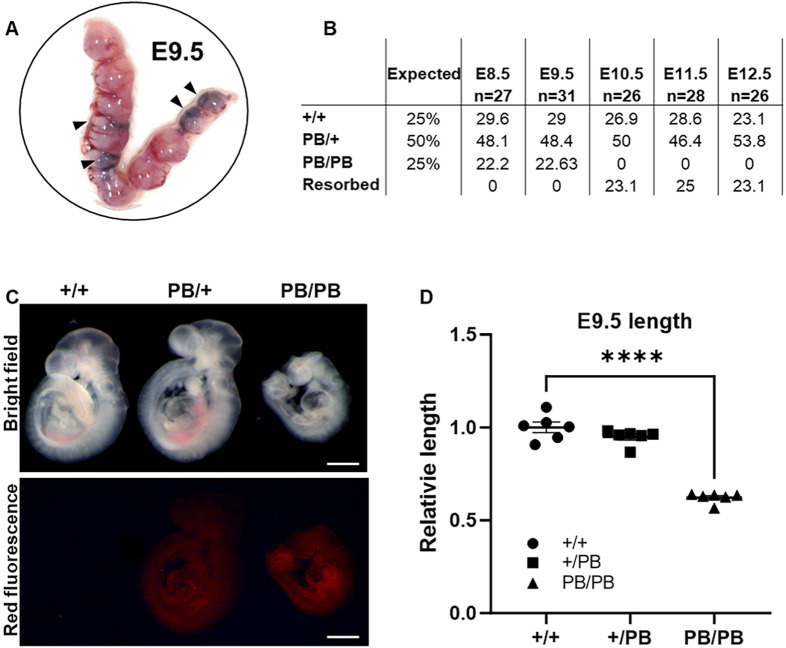
Zmym2 PB mice is homozygous embryo lethal, embryos show developmental delays by E9.5D and lethality from E10.5D to E12.5D. **(A)** Representative photographs of gravid uteri harvested from *Zmym2* PB/+ × *Zmym2* PB/+ pregnancies at E9.5D. Arrowhead denote fetal growth retardation. **(B)** Embryonic viability by genotype from E8.5D to E12.5D compared to the expected Mendelian ratios from *Zmym2* PB/+; *Zmym2* PB/+ crosses. **(C)** Images taken of E9.5D *Zmym2* PB/PB embryos representing fetal growth retardation. Scale bars = 500 μm. **(D)** Relative length of embryos (crown to tail) normalized by litter as a ratio to average *Zmym2* +/+ length. ****, P < 0.0001.

*Zmym2* PB/+ mice were mated and their neonatal offspring were genotyped by PCR amplification on postnatal 7 days. No *Zmym2* PB/PB mice were identified across more than three consecutive generations (Supplementary Figure S1A), indicating that *Zmym2* PB/PB embryos are homozygous lethal. This finding is consistent with a previous study and the Mouse Genome Informatics database (MGI:5706016) (Graham-Paquin et al., 2023). To investigate the role of Zmym2 in embryonic development, embryos were collected at different stages. *Zmym2* PB/PB embryos were present at mendelian ratios until E9.5D (Figure 2A). However, by E10.5D, embryonic resorption was observed, and no *Zmym2* PB/PB embryos were recovered, confirming embryonic lethality (Figure 2B). At E9.5D, most *Zmym2* PB/PB embryos exhibited gross phenotypic abnormalities, and a statistically significant reduction in length was noted (Figures 2C, D). *Zmym2* PB/PB embryos seemly appeared reduction in length at E8.5D, but there was no statistical difference (Supplementary Figure S1C).

In contrast, *Zmym2* PB/+ mice are viable and fertile. We monitored the general condition (weight, fur, activity and appetite) and survival rates among different experimental groups over a period of 12 weeks (Supplementary Figures S1D, E). The weight of *Zmym2* PB/+ mice was normal, and their fur, dietary habits, and activity levels were basically the same as those of *Zmym2* +/+ mice. However, a higher proportion of the *Zmym2* PB/+ than *Zmym2* +/+ mice died throughout the observation period (P60D-P180D).

### 3.3 *Zmym2* PB/+ mice display kidney and urinary malformations, but with normal renal function

**FIGURE 3:**
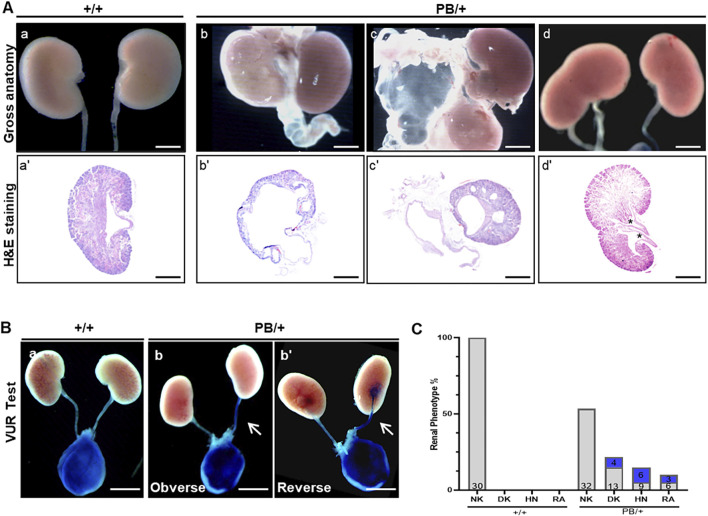
Kidney and urinary tract defects in *Zmym*2 PB mice. **(A)** Overview images and H&E staining of the kidneys of newborn mice. Various urinary malformations observed in *Zmym*2 PB/+ mice. (a, a’) normal kidney; (b, b’) severe hydronephrosis and hydroureter; (c, c’) renal agenesis, and duplex kidney (d, d’). Duplex kidney is defined by double renal pelvises (asterisk). **(B)** Representative images of the vesicoureteral reflux. **(C)** Percentages of various urinary malformations. The numbers in the blue filled boxes represent the number of VUR. The numbers at the bottom of each column of the bar chart represents the total numbers of data in each group. Scale bars, 2 mm in **(A)** (a, b, c, d) and **(B)**; 250 μm in **(A)** (a’, b’, c’, d’). NK, normal kidneys; DK, duplex kidneys; RA, renal agenesis; HN, hydronephrosis; VUR, vesicoureteral reflux.

A comprehensive analysis of the urinary system was performed in 60 newborn *Zmym2* PB/+ mice. Among these, 28 displayed CAKUT phenotypes: 13 (21.7%) had unilateral duplex kidneys (DK), 9 (15.0%) had unilateral hydronephrosis (HN) and 6 (10.0%) had unilateral RA. No significant differences in phenotype distribution were observed between sexes or between left and right kidneys (Figures 3A, C). VUR testing was conducted in all mice (Figure 3B), revealing that unilateral VUR was present in 13 (46.4%) of the 28 CAKUT mice: 4 cases from DK group, 6 cases from HN group, and 3 cases from RA group (Figure 3C). No obvious CAKUT phenotypes were detected in *Zmym2* +/+ mice. Histopathological analysis of the newborn *Zmym2* PB/+ mice was performed. The structure and morphology of the glomeruli and kidney tubules appeared normal in mice with DK. However, in mice with HN, the numbers of glomeruli and tubules were reduced.

To determine whether the *Zmym2* mutation affects kidney function, blood urea nitrogen (BUN) and serum creatinine (Scr) levels were measured. Both BUN and Scr levels remained stable over a 180-day period in *Zmym2* +/+ and *Zmym2* PB/+ mice, with no statistically significant differences between the two groups (Supplementary Figure S2A). Furthermore, H&E staining revealed no structural abnormalities in the glomeruli of either *Zmym2* PB/+ or *Zmym2* +/+ mice (Supplementary Figure S2B).

### 3.4 Morphology of testis and mature epididymal sperm from *Zmym2* PB/+ mice

**FIGURE 4:**
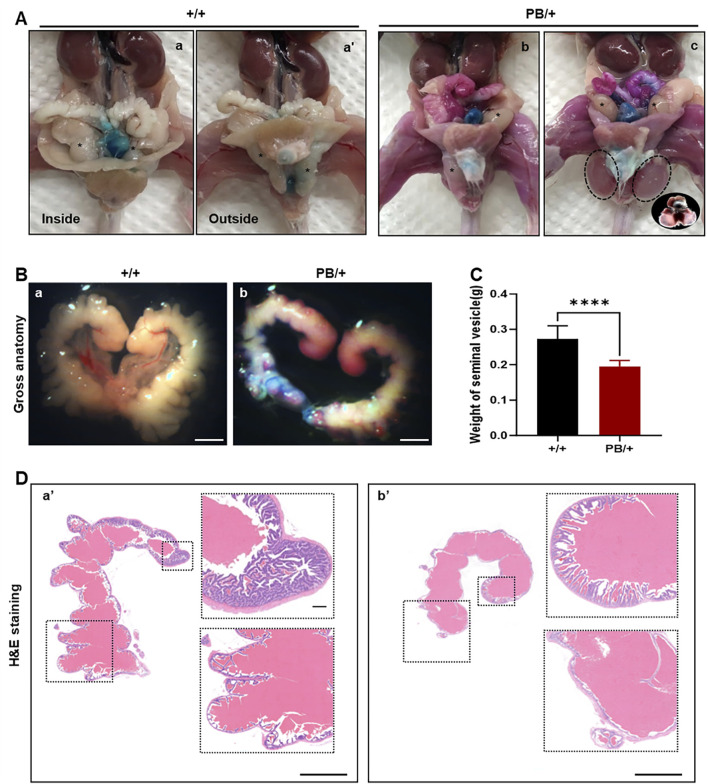
The abnormal manifestations of testis, seminal vesicles and caudal epididymis in *Zmym*2 PB mice. **(A)** Gross anatomy of genital system of adult male mice. (a, a’) normal position of testis (asterisk) in *Zmym2* +/+ mice, unilateral (b) and bilateral (c) cryptorchidism with hydrocele (circle) in *Zmym2* PB/+ mice. **(B)** Gross phenotype of seminal vesicle atrophy and a 32% decrease in seminal vesicle weight in *Zmym2* PB/+ mice **(C)**. **(D)** H&E staining of seminal vesicle. Mucosal folds of seminal vesicle in *Zmym2* PB/+ mice disappeared or reduced compared with *Zmym2* +/+ mice. Scale bars, 2 mm in C (a, b); 250 μm in D (a’, b’).

The morphology of the testis, seminal vesicles, and caudal epididymis was examined. Most male adult *Zmym2* PB/+ mice (77.78%, 7/9) exhibited seminal vesicle atrophy (Figure 4A), with a 32% reduction in seminal vesicle weight compared to *Zmym2* +/+ controls (Figure 4B). Additionally, some *Zmym2* PB/+ mice displayed unilateral cryptorchidism (22.22%, 2/9), bilateral cryptorchidism with hydrocele (11.11%, 1/9) or intratesticular cysts (11.11%, 1/9). The morphology of mature epididymal sperm in *Zmym2* PB/+ mice was normal, with sperm heads exhibiting typical morphology.

To assess the impact of the Zmym2 mutation on sperm quantity and quality, sperm counts were compared between Zmym2 PB/+ and Zmym2 +/+ mice (Table 1). No significant difference in total sperm number in the epididymis. However, the average path velocity (VAP), straight-line velocity (vSL) and curvilinear velocity (vCL) were significantly reduced in *Zmym2* PB/+ mice (p < 0.05). The percentage of progressive mobile sperm (level a and b) was slightly lower in the Zmym2 PB/+ group (p = 0.14), whereas the percentage of sperm with slow velocity (level c) was significantly higher (p < 0.05). These findings indicate that sperm motility is impaired in Zmym2 PB/+ mice due to the Zmym2 mutation.

**TABLE 1:** Comparable motility of cauda epididymal sperm from+/+and PB/+ mice.

CASA parameter	+/+	PB/+	p-value
Total sperm(M)	25.3 ± 3.1	25.3 ± 3.2	0.842
Motile sperm (%)	76.4 ± 5.3	76.9 ± 6.5	0.757
Progressive sperm (%)	71.0 ± 2.7	70.2 ± 0.8	0.077
Path Velocity (VAP) (μm/S)	52.7 ± 0.9	44.4 ± 1.6	0.001
Prog. Velocity (VSL) (μm/S)	35.2 ± 1.2	22.2 ± 2.1	0.001
Track speed (VCL) (μm/S)	106.3 ± 4.0	107.3 ± 4.2	0.788
Lateral amplitude (ALH) (μm)	7.3 ± 1.1	7.4 ± 0.9	0.908
Beat frequency (BCF) (Hz)	30.4 ± 1.7	308 ± 0.7	0.719
Straightness (STR) (%)	67.5 ± 3.9	67.9 ± 3.8	0.889
Linearity (LIN) (%)	34.6 ± 1.5	35.0 ± 1.7	0.774
Elongation (%)	36.8 ± 3.0	37.0 ± 3.0	0.929
Rapid Velocity (level a) (%)	78.9 ± 1.1	68.6 ± 5.0	0.025
Slow Velocity (level b) (%)	20.4 ± 0.6	28.9 ± 3.8	0.018
Static Velocity (level c) (%)	1.4 ± 0.5	2.4 ± 1.2	0.228
b + c (%)	23.7 ± 3.1	16.7 ± 3.2	0.047
Normal morphology (%)	76.1 ± 7.6	54.2 ± 3.7	0.011

Cauda epididymal sperm were incubated in HTF, medium then subjected to CASA. At least 1,000 sperm were examined for each sperm sample (n = 6 for each genotype). Data in sperm analysis are mean ± SD. p < 0.05 was regarded as a significant difference in all parameters between *Zmym2* +/+ and *Zmym2* PB/+ male mice.

Despite the observed morphological abnormalities in the testis, seminal vesicles, and epididymis, as well as changes in sperm motility Zmym2 heterozygosity did not appear to affect male fertility. Fertility was assessed by continuously mating *Zmym2* +/+ and Zmym2 PB/+ males with 8-week-old wild-type females at a 1:2 male-to-female ratio. To control for genetic background differences, females from different strains (S129 and C57BL/6) were used. No significant differences were observed between the two groups in terms of the ability to induce vaginal plugs or achieve pregnancy in wild-type females. Furthermore, the mean litter size was nearly identical in both groups (Supplementary Table S1), indicating that the Zmym2 mutation does not affect vaginal plug formation or pregnancy rate.

### 3.5 *Zmym2* PB/+ mice demonstrated anxiety and aggressive like behaviors with no apparent abnormalities in the craniofacial structure

**FIGURE 5:**
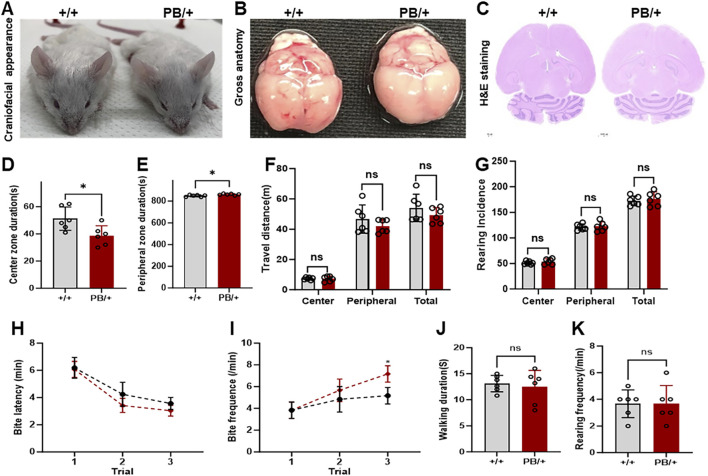
Anxiety and aggressive-like behaviors in *Zmym2* PB/+ mice occur without obvious abnormalities in craniofacial structure. **(A)** General craniofacial appearance observation of adult mice. **(B)** Overview images of whole brain tissue. **(C)** HE staining of the largest coronal section of brain tissue. **(D–G)** Analysis of anxiety. Each mouse was placed in the center of the square (open field) and its behavior was recorded for 15 min. Duration in center zone **(D)** and peripheral zone **(E)**, **(F)** distance traveled and **(G)** incidence of rearings of male adult *Zmym2* PB/+ and *Zmym2* +/+ mice. **(H–K)** Analysis of aggressive behavior. The resident–intruder paradigm was used to assess inter-male aggression. Latency **(H)** and frequency **(I)** of biting attack during 5 min of the first aggressive behavior test. *, p < 0.05, n = 6 for each experimental group, each column and vertical bar represent the mean and standard deviation (SD) in **(D–G, J, K)**. Each symbol and vertical bar represent the mean and SD in **(H, I)**. ns, no significant difference.

The craniofacial morphology, gross brain structure, and histopathology (H&E staining) of brain tissue were examined in *Zmym2* PB/+ and *Zmym2* +/+ mice. No craniofacial abnormalities were detected in either genotype (Figures 5A–C). Behavioral phenotypes were evaluated in *Zmym2* PB/+ mice. Anxiety levels were assessed using the open-field test. Male *Zmym2* PB/+ mice were significantly more active than male *Zmym2* +/+ controls during the first 15 min of the test and spent less time in the center of the chamber (Figures 5D–G) suggesting increased anxiety-like behavior. However, no significant difference in anxiety levels was observed between female *Zmym2* PB/+ and *Zmym2* +/+ mice. Aggressive behavior, was assessed using the resident-intruder paradigm. The latency to the first biting attack did not differ between male *Zmym2* PB/+ and *Zmym2* +/+ mice (Figure 5H). The frequency of biting attacks was similar between genotypes during the first and second trials. However, in the third trial, the frequency of biting attacks was significantly higher in *Zmym2* PB/+ mice than in *Zmym2* +/+ mice (Figure 5I) indicating that *Zmym2* PB/+ mice exhibited increased aggression following repeated encounters with intruders. Locomotor activity was evaluated by recording walking duration and the number of rearings during the first trial. No significant differences were observed between for these measures *Zmym2* PB/+ and *Zmym2* +/+ mice for either measure (Figures 5J, K).

### 3.6 Disturbance in glucose homeostasis of *Zmym2* PB/+ mice

**FIGURE 6:**
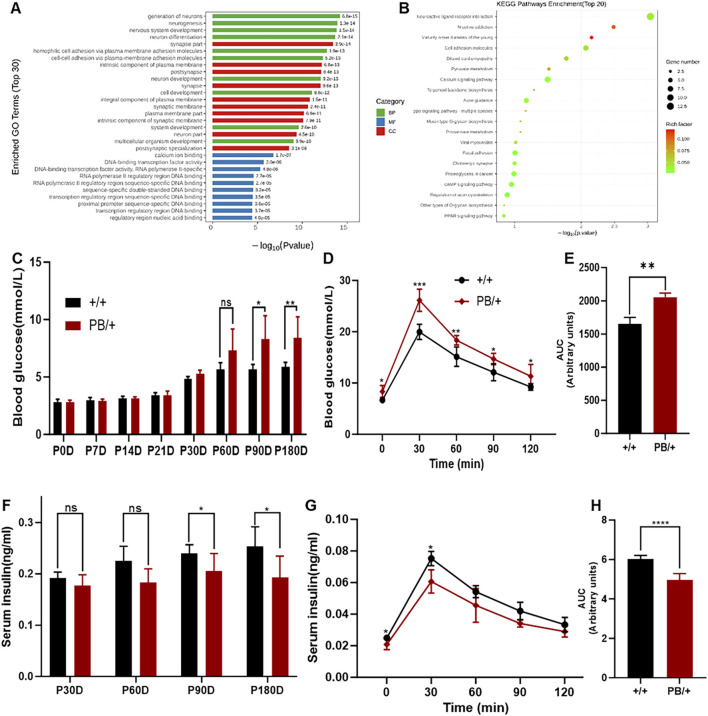
RNA-seq analysis and glucose homeostasis disturbance of *Zmym2* PB/+ mice. **(A)** Gene Ontology (GO) term enrichment analysis. Significantly enriched GO terms were selected based on a Q-value means false discovery rate (FDR) < 0.05. GO terms of the categories of biological processes, cellular components, and molecular functions are depicted in red, green, and blue, respectively. **(B)** Bubble plots of the GO enrichment of DEGs in the E12.5D embryos FDR or adjusted P-value. Rich ratio is the ratio of the signaling pathway of enrichment on the number of differentially expressed genes and signaling pathways of all the genes. **(C)** Random blood glucose of *Zmym2* PB mice at different ages (0, 7, 14, 21, 30, 60, 90 and 180 days). **(D)** Oral glucose tolerance test of 90-day-old mice. **(E)** Area under the glucose curve (AUC) during oral glucose tolerance test. **(F)** Random serum insulin levels of *Zmym2* PB mice at different ages (30, 60, 90 and180 days). Serum insulin concentrations **(G)** and area under the insulin curve **(H)** during oral glucose tolerance test in 90-day-old mice. Data represent mean ± SD. *, p < 0.05; **, p < 0.01; ***, p < 0.001; ****, p < 0.0001; n = 6 for each experimental group. BP, Biological processes; CC Cellular components; MM, Molecular functions.

ZMYM2 plays a critical role in embryogenesis, potentially by regulating gene expression and cellular functions essential for development (Graham-Paquin et al., 2023). To further investigate the role of *Zmym2* in embryonic development and understand the lethality of *Zmym2* PB/PB embryos, mRNA was were extracted from the E9.5D embryos of *Zmym2* +/+ and *Zmym2* PB/PB mice for RNA-sequencing (RNA-seq). The analysis identified 342 DEGs between wild-type and homozygous mutant mice, including 221 upregulated genes and 121 downregulated genes. These DEGs were categorized and enriched for functions related to DNA-bind transcription factor activity, nervous system development and so on. Notably, the most significantly downregulated DEGs associated with *Zmym2* mutation were linked to maturity-onset diabetes of the young (MODY). RNA-seq profiles between *Zmym2* +/+ and *Zmym2* PB/+ embryos highlight the consistent results that MODY associated genes are the most significantly downregulated DEGs (Figures 6A, B).

Subsequent glucose metabolism tests in *Zmym2* PB/+ mice revealed progressive disturbances in glucose homeostasis until 90 days of age, and then stabilizing (Figure 6C). At 90 days, both random and fasting blood glucose levels were significantly elevated in Zmym2 PB/+ mice and remained stable throughout the study. During the OGTT at 90 days, blood glucose concentrations in *Zmym2* PB/+ mice were significantly higher than those in *Zmym2* +/+ mice at all measured time points. The area under the glucose curve (AUC) in 90-day-old *Zmym2* PB/+ mice was significantly greater than that in *Zmym2* +/+ mice (Figures 6D, E). Additionally, random serum insulin levels in *Zmym2* PB/+ mice were significantly lower starting from P90D (Figure 6F). During the OGTT performed with 90-day-old mice, serum insulin levels in *Zmym2* PB/+ mice were significantly elevated at 30 min post-glucose administration (Figure 6G). However, the AUC for insulin in *Zmym2* PB/+ mice was significantly lower than that in *Zmym2* +/+ mice. Between 60 and 90 min after glucose administration, AUC insulin levels in Zmym2 PB/+ mice were comparable to those in Zmym2+/+ mice (Figure 6H). The pancreatic morphology and histological appearance of *Zmym2* PB/+ mice were unchanged compared to those of *Zmym2* +/+ mice (Supplementary Figure S3).

### 3.7 *Zmym2* mutation induces duplicated UB budding and abnormal expression of Tbx18 in kidney

**FIGURE 7:**
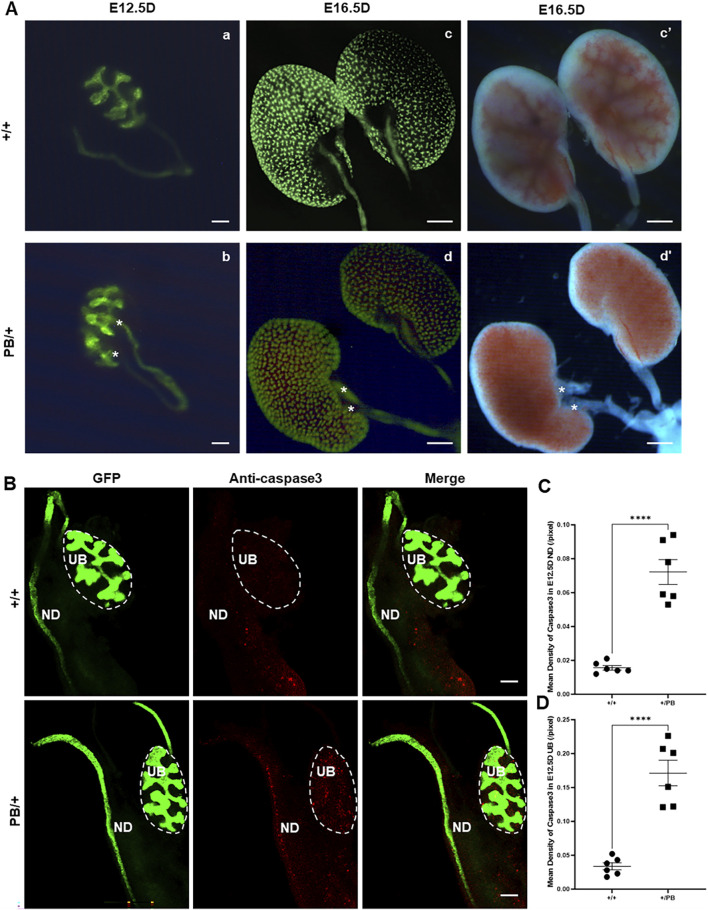
Duplicated UB budding and abnormal apoptosis in *Zmym2* PB/+ kidney Primordia. **(A)** Observation of embryonic kidney development. Two ureteric buds (UBs) formed from one nephric duct (ND) in E12.5D *Zmym2* PB/+ kidney primordia, asterisk shows duplicated UBs (b); unilateral duplicated kidneys accompanied with double ureters (red asterisk) in E16.5D *Zmym2* PB/+ mice (d and d’). **(B)** Anti-Caspase3 staining in UB and ND tissues by immunofluorescence. **(C, D)** Each value of mean density of Caspase3 in E12.5D UB and ND is expressed as the mean ± SD. ****, p < 0.0001. Scale bars, 1 mm in A (c, d, c’, d’); 200 μm in **(A)** (a, b) and **(B)**. UB, Ureteric Bud, ND, Nephric Duct.

Duplex kidney was the most frequently observed urinary system abnormality in *Zmym2* PB/+ mice, occurring in 13 of 28 cases (46.4%). Urinary system malformations originate early in embryonic development, and duplicated UB formation is associated with duplex kidney in mice. To examine the effect of *Zmym2* mutation on UB budding, kidney primordia were isolated at E12.5D from *Zmym2* PB/+ and *Zmym2* +/+ mice. Duplicated UBs were observed in nine of 36 (25.0%) *Zmym2* PB/+ samples, whereas no aberrant UB formation occurred in Zmym2 +/+ mice (Figure 7A).

**FIGURE 8:**
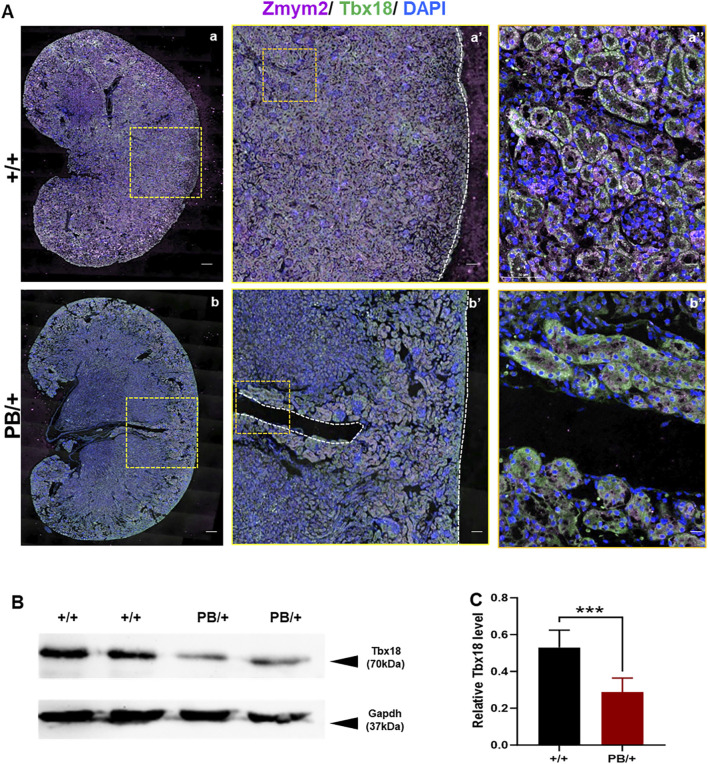
Abnormal expression of Tbx18 in mouse kidney induced by Zmym2 mutant related to mouse kidney development. **(A)** Immunofluorescence staining of Zmym2 and Tbx18 in the renal tissue of *Zmym2* PB mice. Full image of kidney in a, b; focused observation of kidney nephrogenic zone in a’, b’, white dashed line indicates nephrogenic zone; enlarged image to clearly depict subcellular localization in a’’, b’’. Scale bars, 200 μm in a, b; 50 μm in a’, b’; 10 μm in a’’, b’’. **(B, C)** Western blots showed that the Tbx18 protein expression in kidney tissues was reduced approximately 50% in *Zmym2* PB/+ group, compared to *Zmym2* +/+ group. ***, p < 0.001.

Defects in apoptosis can contribute to various CAKUT phenotypes. Apoptosis in the UB is crucial for normal UB growth and subsequent branching morphogenesis. A wave of apoptosis in the ND that is likely necessary for separating the ureters from the ND (Mendelsohn et al., 2009). The number of apoptotic cells in the ND and UB was compared, revealing a significant increase in caspase3 positive apoptotic cells in these regions in *Zmym2* PB/+ mutants (Figures 7B–D), which may contribute to the development of VUR and other CAKUT phenotypes. Previous proteomic analyses identified ZMYM2 as endogenous binding partner of TBX18 in 293 and A549 cells. Tbx18 is co-expressed with Zmym2 in the mesenchymal compartment of the developing ureter in mice, and mutations in TBX18 and in ZMYM2 have recently been linked to CAKUT, supporting the functional relevance of TBX18 and ZMYM2 interaction in ureter development (Ludtke et al., 2022). To investigate the effect of *Zmym2* mutation on Tbx18 expression in kidney, immunofluorescence staining and western blot were performed to assess co-localization and expression changes in the kidney tissue. Zmym2 and Tbx18 were primarily co-localized in kidney interstitial cells, with particularly high expression in the nephrogenic zone(Figure 8A). *Zmym2* PB/+ kidneys exhibited discontinuous nephrogenic zones, which was consistent with the formation of multiple UBs. In comparing to *Zmym2* +/+ group, Tbx18 expression in *Zmym2* PB/+ kidney tissue was decreased approximately 50% (Figures 8B, C).

## 4 Discussion

In our previous study (Connaughton et al., 2020), a mouse model was generated to replicate the frameshift mutation identified in exon 3 of patient GM1-21 (p. Gly257* [c.766_767dupGT]) using CRISPR-Cas9 gene targeting approach. *Zmym2* +/− mice exhibited a spectrum of CAKUT-like defects, including hydroureter, duplex and cystic kidneys, and VUR. Immunofluorescence analysis of E18.5D kidneys without overt malformations showed normal nephrogenesis and branching morphogenesis in *Zmym2* +/− animals. No additional phenotypes were observed in *Zmym2* +/− mice. In this study, *Zmym2* PB mice was successfully generated using PB transposon-based insertional mutagenesis, revealing that *Zmym2* was truncated following PB insertion. *Zmym2* PB mice exhibited multiple developmental malformations of the urinary system predominantly presenting as duplex kidneys. Duplicated UB formation during early metanephric development and abnormal extension of the nephrogenic zone deep into the kidney contributed to the formation of duplex kidneys. In addition to the urinary system defects, *Zmym2* PB/+ mice exhibited developmental abnormalities in the reproductive, nervous, endocrine, and other systems, encompassing a broad spectrum of syndromic CAKUT phenotypes observed in clinical cases. The genetic, clinical, and pathomorphological characteristics of the *Zmym2* PB mice were specified, making this a unique and valuable animal model to studying syndromic CAKUT pathogenesis, prognosis and outcomes. To our knowledge, this is the first *Zmym2* mutant mouse model in which a single -gene mutation resulted in a wide range of syndromic CAKUT phenotypes similar to those observed in human carrying *ZMYM2* mutations. Furthermore, this is first reported *Zmym2* truncated mutant mouse model in literature, highlighting both embryonic lethality and abnormalities in kidney primordia.

ZMYM2 is a newly identified factor involved in DNA methylation patterning in early embryonic development (Graham-Paquin et al., 2023). *Zmym2* PB homozygous mutants are embryonic lethal, while *Zmym2* PB/+ mice are viable and fertile, and capable of normal growth. However, a small proportion of the *Zmym2* PB/+ mice died after P60D, with 10.7% of these mice dying by P180D. At the time of death, no significant deterioration in kidney function was observed suggesting that extra-renal factors may be the primary cause. To further investigate the role of *Zmym2* in embryonic development and lifespan, RNA-Seq was performed on E9.5D embryos from *Zmym2*+/+ and *Zmym2* PB/PB groups. RNA-Seq data provided unexpected findings, revealing a direct mechanistic link between Zmym2 mutation and MODY. Similar to clinical observations in MODY patients, *Zmym2* heterozygous mutant mice exhibited chronic but mild hyperglycemia, with blood glucose levels progressively increasing until 90 days of age. Additionally, these mutant mice displayed significantly reduced fasting serum insulin concentrations and diminished insulin secretory capacity. Due to the stable and mild phenotype, life expectancy was not affected in heterozygous mutant mice before P180D, whereas homozygous mutation was lethal by E10.5D. These findings suggest that some phenotypes may develop progressively with age, emphasizing the importance of considering patient age to improve the diagnostic rates, particularly in younger children. The discovery of HNF1β gene mutations as a cause of developmental kidney disease originally emerged from studies on MODY. *HNF1B* mutations are associated with CAKUT, a slowly progressive decline in kidney function, pancreatic abnormalities, diabetes, and neurological deficits, among other phenotypes (Clissold et al., 2015). Investigating the genetics and molecular pathways of ZMYM2 is essential for understanding both renal and extra-renal phenotypes, as well as for identifying potential areas for future research on ZMYM2-associated diseases, particularly those involving signaling molecule defects.

Sperm motility is a key parameter in assessing normal semen quality, and men with impaired sperm motility have a reduced likelihood of fertilization, which can lead to infertility. In addition to pathological factors such as radiation and oxidative stress, genetic disorders significantly contribute to sperm dysfunction. *ZMYM2* mutations have been linked to abnormal development of the male reproductive system, and the *de novo* or mosaic occurrence of these mutations strongly suggest that heterozygous truncating mutations in *ZMYM2* convey infertility or interfere with germline transmission (Connaughton et al., 2020). In this study, *Zmym2* mutations resulted in structural abnormalities in the male reproductive system and reduced sperm motility, however, fertility was not significantly affected. Several factors may account for the discrepancies between human clinical findings and our results (Mao et al., 2016). Notably, genetic background related to reproductive functions differ significantly between humans and mice. Mice generally exhibit higher reproductive capacity than humans. Additionally, men with *ZMYM2* mutation may be more susceptible to pathological factors and lifestyle influences, including other genetic disorders, genital tract infections, environmental pollutants, and smoking. Although *Zmym2* mutations alone did not induce male infertility in mice, they led to structural abnormalities in the reproductive system and impaired sperm motility, suggesting that *ZMYM2* may be one of the genes involved in male infertility. However, the molecular and cellular mechanisms underlying the effects of ZMYM2 mutations remain unclear and warrant further investigation.

Neurological manifestations including microcephaly, developmental delay, intellectual disability, speech delay and infantile hypotonia, have been reported in CAKUT patients carrying heterozygous nonsense or frameshift mutations of *ZMYM2*. In X. tropicalis, *Zmym2* knockdown of leads to craniofacial defects. However, our findings indicate that heterozygous *Zmym2* mutations on the FVB/N genetic background do not result in detectable craniofacial abnormalities. This finding is unexpected, as it contrasts with previous studies in X. tropicalis, where craniofacial dysmorphology was reported following morpholino-based Zmym2 knockdown. The observed differences may be attributed to the methods used to deplete Zmym2. Morpholinos targeting Zmym2 could potentially affect homologous family members, leading to an exacerbated phenotype. Additionally, species-specific differences and genetic background variations between X. tropicalis and mice may contribute to the discrepancies (Walsh et al., 2017). Despite extensive analyses, no craniofacial abnormalities were identified in *Zmym2* PB mice on the FVB/N background. Notably, microcephaly in patients with *ZMYM2* mutations is not fully penetrant, and the severity of intellectual disability varies among individuals, suggesting that other environmental or genetic factors may influence disease. Future studies involving backcrossing *Zmym2* mutant mice onto different genetic backgrounds may help elucidate this hypothesis. Even in the absence of obvious craniofacial abnormalities, *Zmym2* PB/+ mice exhibited behavioral abnormalities, including anxiety and aggressive-like behaviors, uncovering a previously unrecognized neurologic phenotype.

*ZMYM2* loss-of-function mutations can cause CAKUT, however, the underlying mechanisms of embryonic kidney developmental abnormalities remain unclear and require further investigation. *Zmym2* was highly expressed in kidney primordia from E9.5D, with peak expression E13.5D. confirming its critical role in kidney development (Supplementary Figure S3A). Duplicated UB formation in *Zmym2* mutants was a primary cause of duplex kidney formation, and the reduced apoptosis at the ND in *Zmym2* mutants may have contributed to abnormal ureter-bladder connections, leading to VUR. The transcription factor TBX18 regulates patterning and differentiation programs in the primordia of urinary system. Tbx18 is co-expressed with Zmym2 in the mesenchymal compartment of the developing ureter in mice, and both genes are co-expressed in the ureteric mesenchyme at E12.5D, the stage at which Tbx18 is required for tissue specification (Ludtke et al., 2022). Our study confirmed that Zmym2 and Tbx18 were co-localized in kidney tissues, with notably high expression in the nephrogenic zone. Tbx18 expression was reduced in kidney tissues of *Zmym2* PB/+ mice, which brought to light that ZMYM2 interacts with TBX18 during the kidney development. Patients carrying *ZMYM2* mutations also exhibit extra-renal abnormalities, including cardiac septal defects, skeletal abnormalities, hypoplastic hands, feet and nails, as well as scoliosis (Connaughton et al., 2020). Although these phenotypes were not observed in *Zmym2* PB/+ mice, *in situ* hybridization and IF analyses demonstrated extensive *Zmym2* expression in multiple organs at E13.5D and prominently in brain, heart, lung, pancreas, kidney and genital tissues at P0D (Supplementary Figures 3B, C). This suggests that Zmym2 plays essential roles in multiple organs systems. Interestingly, *Tbx18*-deficient mice exhibit vertebral column malformations, while heterozygous loss of another TBX family member, Tbx20, results in a range of cardiac defects. These findings raise the possibility that ZMYM2 interacts with TBX18 and other TBX family at various developmental sites to regulate transcriptional repression. Further *in vivo* genetic interaction and molecular studies are necessary to test this hypothesis (Ludtke et al., 2022).

## 5 Conclusion

This study provides the first comprehensive characterization of phenotypes in *Zmym2* mutant mice with the PB insertion, including embryonic lethality, genitourinary defects, anxiety and aggressive-like behavior, and glucose metabolism disorders. Notably, this study offers new insights into the roles of Zmym2 in embryonic development and identifies a potential link between *Zmym2* mutation and MODY, as well as a possible relationship between hyperglycemia and lifespan. Additionally, *Zmym2* mutations induced duplicated UB formation at the early kidney development stage, and abnormal extension of the nephrogenic zone deep into the kidney are the main causes of duplex kidney formation. Reduced apoptosis in the nephric duct may have contributed to abnormal ureter-bladder connections, leading to VUR. The co-expression of Tbx18 with Zmym2 in the developing kidney and reduction in Tbx18 expression in *Zmym2* mutants further support the hypothesis that *Zmym2* interacts with Tbx18 during kidney development.

In summary, this study expands the *Zmym2* genotype-phenotype spectrum. The *Zmym2* PB mouse model is the first to demonstrate roles of *Zmym2* in neuroethology and endocrinology, extending its significance beyond genitourinary defects and embryonic development. Further investigation of these phenotypes in CAKUT patients carrying *ZMYM2* mutations will enhance our understanding of the disease and improve strategies for early diagnosis, monitoring, and treatment.

## Data Availability

The datasets presented in this study can be found in online repositories. The names of the repository/repositories and accession number(s) can be found in the article/Supplementary Material.
