# Supplementary material for: Genitourinary defects, anxiety and aggressive-like behavior and glucose metabolism disorders in Zmym2 mutant mice with inserted piggyBac transposon
Source: Front Cell Dev Biol. 2025 Apr 17;13:1523266. doi: 10.3389/fcell.2025.1523266 (PMC12043690; doi:10.3389/fcell.2025.1523266)
Supplement: Supplementary file 1 [file Presentation1.zip › Supplementary methods and figure legends/Supplementary Figure Legends.docx]

**Supplementary Figure legends**

**Supplementary Figure 1.** ***Zmym2* PB/+ mice are viable and fertile. (A)** The number of offspring mice from *Zmym2* PB/+ × *Zmym2* PB/+ cross. **(B)** Genotype PCR of *Zmym2* PB mice. **(C)** Gross view of embryos at E8.5D in *Zmym2* +/+, *Zmym2* PB/+ and *Zmym2* PB/PB groups. **(D)** Body weight of *Zmym2* +/+ and *Zmym2* PB/+ mice (both n=30) from P0D to P120D, housed in the same cage types. Data represent means and SD. **(E)** Survival percentage of *Zmym2* +/+ and *Zmym2* PB/+ mice at different ages (0, 30, 60, 90, 120, 150 and180 days).

**Supplementary Figure 2. Kidney function and multi-organ tissue structure of *Zmym2* PB mice. (A, B)** Time courses of blood urea nitrogen level and serum creatinine level of *Zmym2* +/+ mice and *Zmym2* PB/+ mice. **(C)** H&E staining kidney sections harvested at different time points (8 weeks and 12 weeks) from *Zmym2* +/+ and *Zmym2* PB/+ mice. **(D)** H&E staining sections of the heart, lung, liver, pancreas and spleen of *Zmym2* PB mice.

**Supplementary Figure 3. Spatiotemporal analysis of *Zmym2* expression. (A)** *Zmym2* mRNA expression in kidney primordia and in mature kidneys at different stages of development. **(B)** *Zmym2* mRNA expression in muti-organ tissues from P0D mouse embryos. **(C)** *Zmym2* mRNA expression in situ hybridization in E13.5D mouse embryos sections covers all major organ systems. Bn, Brain; Ht, Heart; Lu, Lung; Li, Liver; In, Intestine; Kd, Kidney. Scale bars =2000um.
