# Supplementary material for: Genitourinary defects, anxiety and aggressive-like behavior and glucose metabolism disorders in Zmym2 mutant mice with inserted piggyBac transposon
Source: Front Cell Dev Biol. 2025 Apr 17;13:1523266. doi: 10.3389/fcell.2025.1523266 (PMC12043690; doi:10.3389/fcell.2025.1523266)
Supplement: Supplementary file 1 [file Presentation1.zip › Supplementary methods and figure legends/Supplementary Methods.docx]

**Supplementary Methods**

##### Western blot

Western blotting was performed according to the standard procedures. An equal amount of each protein lysate was loaded onto 8% or 10% sodium dodecyl sulfatepolyacrylamide gel electrophoresis gels and blotted onto polyvinylidene fluoride membranes. Samples were blocked in Tris-buffered saline Tween 20 (20 mM Tris-HCl, pH 7.4 and 0.05% Tween 20) with 5% non-fat dry milk. The membranes were incubated with primary antibodies, mouse monoclonal antibody to glyceraldehyde-phosphate dehydrogenase (GAPDH, 1:1000) or rabbit polyclonal antibody to Zmym2 (Abcam, #ab106624, 1:1000) at appropriate dilutions overnight at 4°C and horseradish peroxidase-linked secondary antibody (at a dilution of 1:2000) (Santa Cruz, # sc-2354, 1:2000) for 1 hour at room temperature. The results were visualized by fluorography using the Tanon gel imaging system (Tanon, Shanghai, China).

##### VUR experiments

Operators who were blinded to the genotypes performed the VUR test. Mice were dissected using an anterior midline incision to expose the kidneys and the urinary tract. The bladder was punctured with a 25gauge needle to manually inject methylene blue (1 mg/ml in PBS) at a rate of 100 μl/min until the dye exited through the urethra. The severity of VUR was determined by the extent of the ureter dilation as our previous study(1).

##### Histopathological analysis

Kidneys with attached ureters and bladders and other organs were removed from the euthanized mice. Tissues were fixed in 4 % paraformaldehyde, embedded in paraffin, sectioned at 4μm, stained with hematoxylin and eosin (H&E) and examined under a light microscope as previously described(1).

##### RNA in situ hybridization

Whole-mount in situ hybridization was performed as previously described (2). E13.5D embryos tissues were placed in fixation solution (prepared with DEPC water). Once fixation is complete, dehydration, sectioning, deparaffinization, rehydration, digestion, blocking endogenous peroxidases were followed. After pre-hybridization, the hybridization solution containing *Zmym2* probe (5’-DIG-CGCCACGCCACTGCACTCGCTCTTT-DIG-3’) at a concentration of 8 ng/µl. Incubate overnight at 37°C in a temperature-controlled incubator, then performed post-hybridization washing, adding blocking solution, adding anti-Digoxigenin-HRP, DAB staining, counterstaining the nucleus, dehydration and mounting. Microscopic examination and analysis were done finally.

#### References

1. Liu J, Sun L, Shen Q, Wu X, Xu H. New congenital anomalies of the kidney and urinary tract and outcomes in Robo2 mutant mice with the inserted piggyBac transposon. BMC Nephrol. 2016;17(1):98.

2. Wang H, Zhang C, Wang X, Lian Y, Guo B, Han M, et al. Disruption of Gen1 Causes Congenital Anomalies of the Kidney and Urinary Tract in Mice. Int J Biol Sci. 2018;14(1):10-20.
