# Supplementary material for: Genitourinary defects, anxiety and aggressive-like behavior and glucose metabolism disorders in Zmym2 mutant mice with inserted piggyBac transposon
Source: Front Cell Dev Biol. 2025 Apr 17;13:1523266. doi: 10.3389/fcell.2025.1523266 (PMC12043690; doi:10.3389/fcell.2025.1523266)
Supplement: Supplementary file 2 [file Table1.docx]

**Supplementary Table 1. Targeted disruption of Zmym2 did not affect male fertility in mice**

| Genetic background | Male mice | Female mice | Plugged mice | Pregnant mice | Offspring  (M/F) | AOA | FCP(%) | FC(%) |
| --- | --- | --- | --- | --- | --- | --- | --- | --- |
| C57BL/6 | PB/+ (n=6) | +/+ (n=12) | 9 | 6 | 52 (27:25） | 8.67 | 75.00 | 50.00 |
|  | +/+ (n=6) | +/+ (n=12) | 10 | 7 | 45 (21:24） | 6.43 | 83.33 | 58.33 |
| S129 | PB/+ (n=6) | +/+ (n=12) | 8 | 7 | 38 (18:20) | 5.42 | 66.67 | 58.33 |
|  | +/+ (n=6) | +/+ (n=12) | 10 | 6 | 42 (16:26) | 7.00 | 83.33 | 50.00 |
| FVB/N | PB/+ (n=6) | +/+ (n=12) | 10 | 9 | 60 (20:40) | 6.67 | 83.33 | 75.00 |
|  | +/+ (n=6) | +/+ (n=12) | 9 | 8 | 58 (26:32) | 7.25 | 75.00 | 66.67 |

Average offspring amount (AOA) was calculated as the total number of offspring divided by the number of females that gave birth to offspring. Frequency of copulatory plug (FCP) was calculated as the ratio of the number of plugged females to total number of females to which males with the same genotype had access. Frequency of conception (FC) was calculated as the ratio of the number of females that gave birth to offspring to total number of females. p>0.05 for all the comparison of FCP or FC. M, male; F, female.
