# Supplementary figures and images for: Genitourinary defects, anxiety and aggressive-like behavior and glucose metabolism disorders in Zmym2 mutant mice with inserted piggyBac transposon
Source: Front Cell Dev Biol. 2025 Apr 17;13:1523266. doi: 10.3389/fcell.2025.1523266 (PMC12043690; doi:10.3389/fcell.2025.1523266)

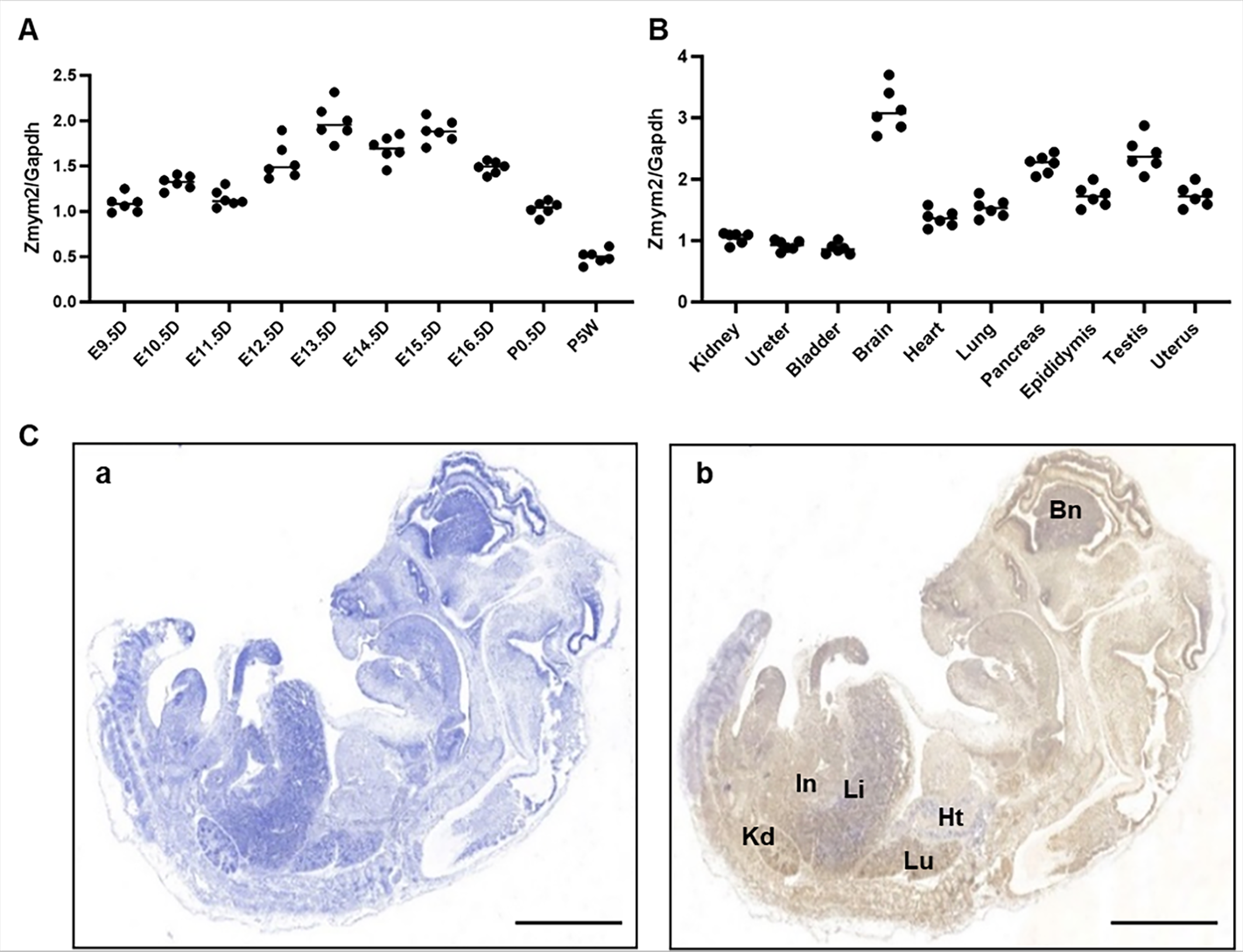

Supplement: Supplementary file 3 [file Image3.tif]

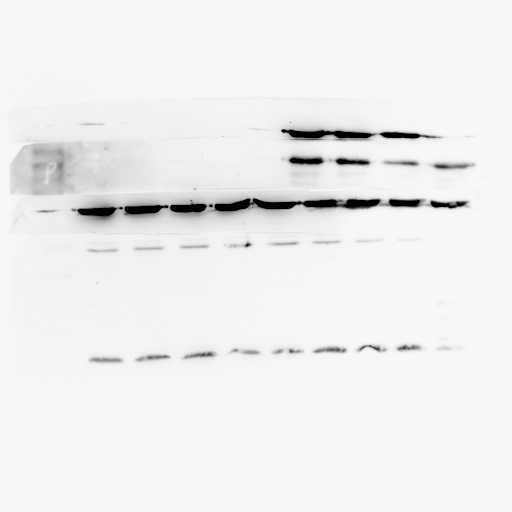

Supplement: Supplementary file 4 [file Image4.tif]

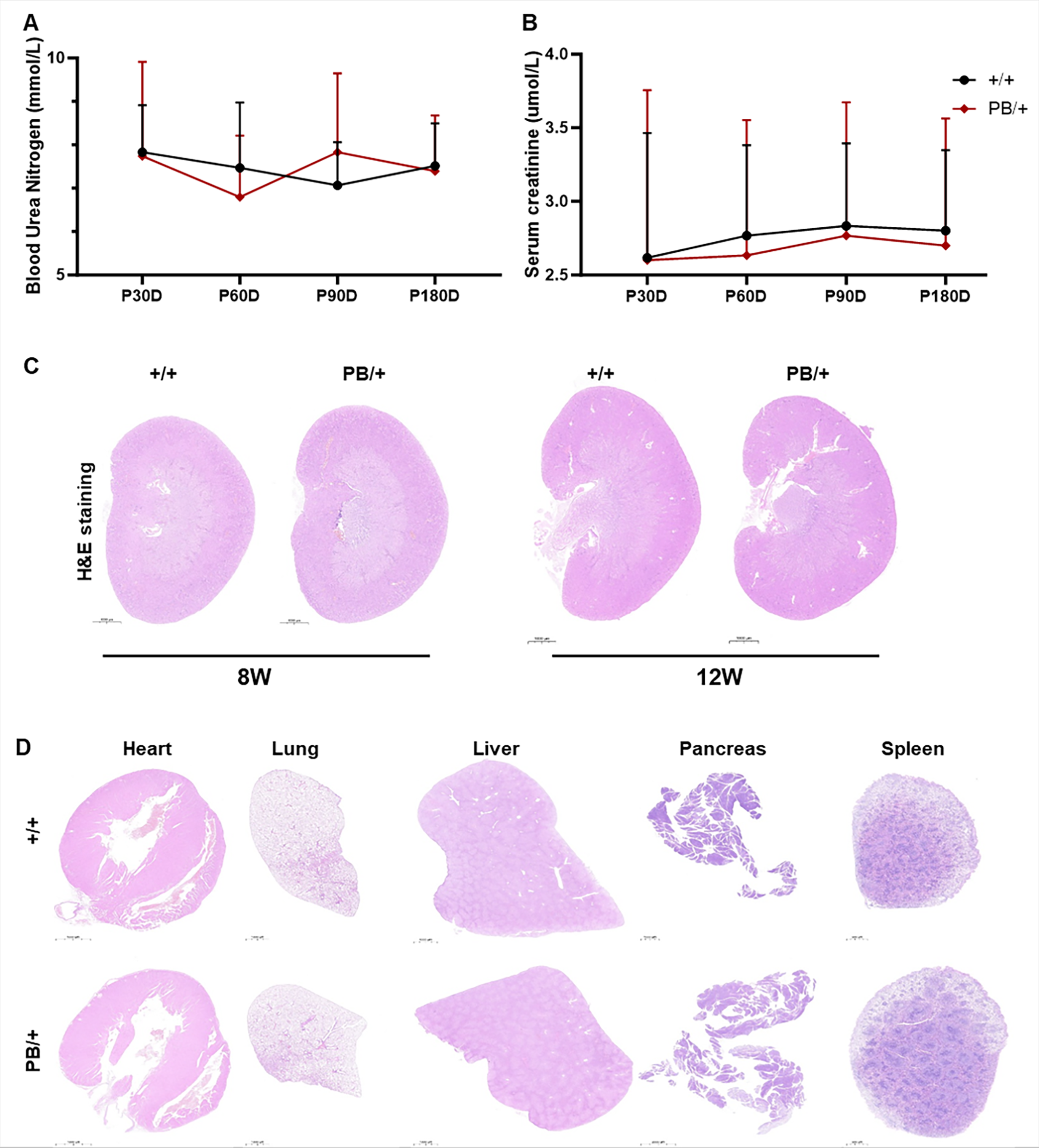

Supplement: Supplementary file 5 [file Image2.tif]

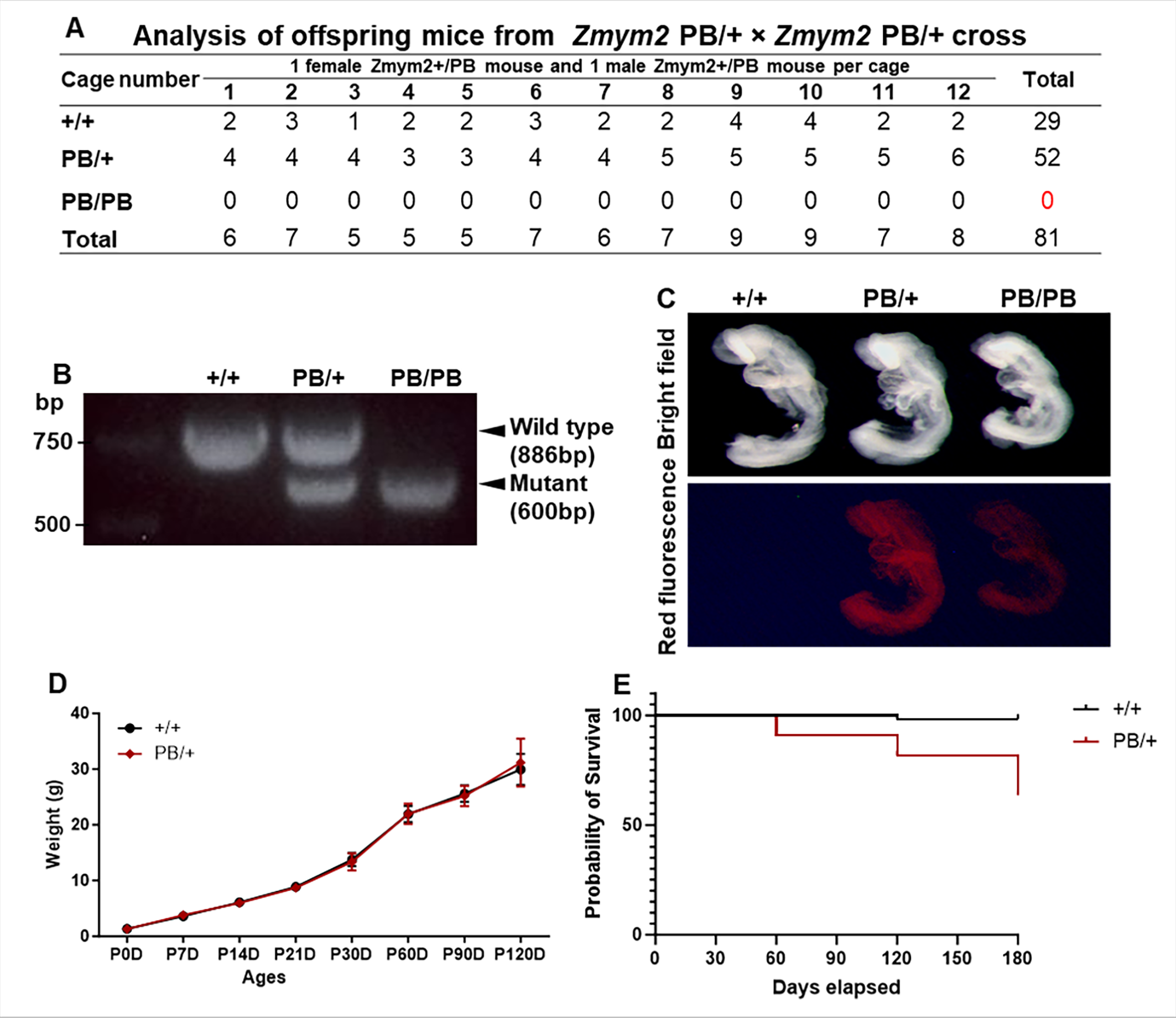

Supplement: Supplementary file 6 [file Image1.tif]
